# Diagnostic accuracy of serum dickkopf-1 protein in diagnosis hepatocellular carcinoma: An updated meta-analysis

**DOI:** 10.1097/MD.0000000000016725

**Published:** 2019-08-09

**Authors:** Zhenjie Li, Lisha Mou, Haibin Gao, Yi Zeng, Xueyi Tang, Xuesong Deng, Zuhui Pu, Yong Ni, Yongqiang Zhan

**Affiliations:** a Department of General Surgery, The Second People's Hospital of Shenzhen, Shenzhen; b Shantou University Medical College, Shantou; c Department of Central Laboratory, The Second People's Hospital of Shenzhen; d Department of General Surgery, The People's Hospital of Longhua Shenzhen; e Department of Radiology, The Second People's Hospital of Shenzhen, Shenzhen, China.

**Keywords:** diagnose, hepatocellular carcinoma, meta-analysis

## Abstract

**Background:**

To verify the accuracy of serum dickkopf-1 protein (DKK-1) in the diagnosis of hepatocellular carcinoma (HCC) by an updated meta-analysis.

**Methods:**

We searched potential eligible studies in PubMed and Embase before July 8, 2018. Sensitivity (SN), specificity (SP), positive likelihood ratio (PLR), negative likelihood ratio (NLR), summary receiver operating characteristics curve (sROC), and diagnostic odds ratio (DOR) were pooled with their 95% confidence intervals CIs) using a bivariate random-effects model.

**Results:**

A total of 8 articles contained 10 studies on diagnosis of HCC with DKK-1 alone,7 articles contained 9 studies on diagnosis of HCC with a-fetoprotein (AFP) alone and 5 articles contained 7 studies on diagnosis of HCC with DKK-1 + AFP were identified. The pooled SN, SP, PLR, NLR, and DOR of DKK-1 alone, AFP alone and DKK-1 + AFP were 0.72 (95% CI: 0.70–0.75), 0.62 (95% CI:0.59–0.64) and 0.80 (95% CI:0.78–0.83), 0.86 (95% CI: 0.84–0.87), 0.82 (95% CI:0.80–0.84) and 0.87 (95% CI: 0.85–0.88), 4.91 (95% CI: 2.73–8.83), 3.60 (95% CI:2.01–6.44) and 6.18 (95% CI: 4.68–8.16), 0.32 (95% CI: 0.22–0.47), 0.49 (95% CI:0.40–0.60) and 0.20 (95% CI: 0.15–0.26), and 17.21 (95% CI: 9.10–32.57), 7.45 (95% CI:3.69–15.01) and 31.39 (95% CI: 23.59–43.20), respectively. The area under the sROC was 0.88, 0.70, and 0.92 for the 3 diagnostic methods.

**Conclusions:**

Serum DKK-1 + AFP showed a high accuracy for diagnosis of HCC, and serum DKK-1 alone had moderate accuracy as compared to a previous meta-analysis, while AFP alone owned an unsatisfied diagnostic behavior for HCC. Due to the limitations of the current analysis, further well-designed studies are needed to confirm the diagnostic value of DKK-1 and DKK-1 + AFP in HCC diagnosis.

## 1 Introduction

Hepatocellular carcinoma (HCC) is one of the most common malignant tumors, with about 78,200 newly diagnosed cases per year and second highest mortality rate worldwide.^[1,2]^ Its incidence is expected to increase in the next 10 to 20 years. The 5-year survival rate differs by stages, with the rate of 50% to 75% in the early stage, which further decreases to 3% for distant metastasis HCC patients.^[3,4]^ Hepatitis B/C virus infection, alcohol, nonalcoholic fatty liver disease, Budd-Chiari syndrome, aflatoxin, and so on, were identified as risk factors for HCC. In clinical practice, serum a-fetoprotein (AFP) and ultrasonography are widely utilized for early detection of HCC.^[5]^ However, with a sensitivity (SN) of 53% and specificity (SP) of 90% at a cut-off value of 20 ng/ml, western countries have excluded AFP for HCC diagnosis due to its lack of accuracy.^[6–8]^ Furthermore, AFP-negative HCC could be missed if it is used as a marker for diagnosis of HCC.

Surgery, local treatment, radiation therapy, systemic therapy, and so on, are currently used in the management of different stages of HCC, but there are limitations for clinical application of surgery and nonsurgical treatments are incapable of significantly improving overall survival and avoiding relapse of HCC.^[4,9]^ Current methods for early screening of HCC include imaging and tumor biomarkers.^[10,11]^ Circulating cell-free nucleic acids could also contribute to the diagnosis of HCC.^[12]^ Among these methods, biomarkers seem to be more convenient and cost-effective.

Dickkopf-1 protein (DKK-1) was first identified in 1998 and plays a key role in head-inducing/head embryogenesis of Xenopus.^[13]^ As a secreted glycoprotein, dysregulated expression of DKK-1 was found in many malignant tumors, such as HCC, pancreatic cancer, colorectal cancer, multiple myeloma, and chronic lymphocytic leukemia.^[14–17]^ Subsequent studies showed that through competing with Wnt ligand, DKK-1 acts as an antagonist of the Wnt signaling pathway, and has been proverbially involved in tumorigenesis, metastasis, recurrence, and poor prognosis of HCC.^[18–21]^ It was also significantly correlated with the tumor size, and concentrations of serum DKK-1 rapidly decreased after resection of HCC.^[22,23]^ Meanwhile, elevated serum DKK-1 level was found in AFP-negative HCC.^[22–25]^

The diagnostic value of serum DKK-1 for HCC had been previously reported.^[22–31]^ A meta-analysis of 4 studies was conducted in 2014 to estimate the exact accuracy of serum DKK-1 for diagnosing HCC.^[32]^ Since several studies have been published in recent years, it is worthwhile to conduct an updated meta-analysis to better understand the diagnostic value of DKK-1 for detecting HCC.

## 2 Methods

The present study was carried out based on the published studies. Thus, the approval from an ethics committee or institutional review board was not required.

### 2.1 Search strategy

This systematic review was conducted based on the preferred reporting items for systematic reviews and meta-analyses guidelines.^[33]^ Relevant articles published in English were searched in PubMed and Embase before July 8, 2018. The search terms used were “hepatocarcinoma or hepatoma or liver cancer or hepatocellular carcinoma or HCC,” “dickkopf-1 or DKK-1.” The reference lists of all relevant articles were manually searched for additional eligible studies. The search procedure was conducted by 2 independent investigators.

The inclusion criteria were:

(1) the study used DKK-1 as a biomarker to diagnose HCC;
(2) the sample type was serum DKK-1;
(3) the diagnosis of HCC was established by pathological methods or in line with correlated accepted guidelines;
(4) the study provided sufficient data to calculate the SN and SP of DKK-1.

The exclusion criteria were:

(1) review articles, meeting reports, comments, or abstracts;
(2) nonhuman studies;
(3) the papers with duplicate patient populations.

### 2.2 Data extraction

Data extraction was performed by 2 independent investigators (Xueyi Tang and Yi Zeng), and any disagreements were resolved by a third author (Yongqiang Zhan). The data extracted from each study included first author, date of publication, geographical region, study design, reference standards, measuring methods and cut-off values, gender and sex ratio of HCC patients, and the number of true positive, false positive, false negative, and true negative subjects.

### 2.3 Study quality assessment

The assessment tool quality assessment of diagnostic accuracy studies 2 (QUADAS-2), which was developed based on QUADAS, was used to assess the quality of each paper.^[34,35]^ QUADAS-2 has 4 domains: patient selection, index test, reference standard, flow, and timing. Each domain of QUADAS-2 was assessed as “yes,” “no” or “unclear.” Signaling questions were used to judge risk of bias as “high” or “low.” A third author (Zuhui Pu) was consulted for any disagreements.

### 2.4 Statistical analysis

Two independent investigators (Xueyi Tang and Yi Zeng) performed the statistical analysis using MetaDisc version 1.4, Revman version 5.3 and STATA version 12.0 software programs, and *P* < .05 represents statistical significance. SN, SP, positive likelihood ratio (PLR), negative likelihood ratio (NLR), and diagnostic odds ratio (DOR) were pooled with their 95% confidence intervals (CIs). Substantial heterogeneity, a nonuniformity indicator, was demonstrated as *I*^2^ value > 50%,^[36]^ and a random-effects model was adopted. The DOR was also pooled since it is an independent factor calculated from PLR and NLR to indicate the performance of diagnosis test. The pooled diagnostic SN, SP, and heterogeneity were demonstrated by forest plots. Summary receiver operating characteristic curves (sROC) represented the total diagnostic efficacy of DKK-1. Threshold effect was evaluated by calculating the Spearman correlation coefficient and *P* < .05 indicated threshold effect.^[37]^ If heterogeneity was not found by threshold effect, subgroup analysis was used for further exploration. Deeks’ funnel plot asymmetry test was utilized for assessing publication bias,^[38]^ and a SN analysis was also performed.

## 3 Results

### 3.1 Study selection

**Figure 1:**
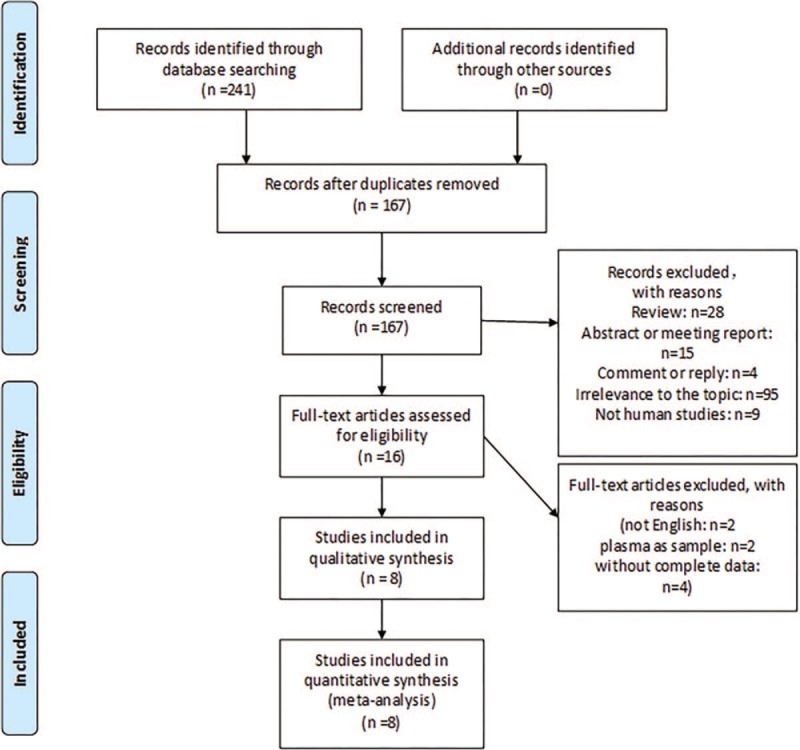
Flow diagram of the study searching.

A total of 241 articles aggregately related to the search terms were retrieved from Medline and EMBASE. After scanning the titles and abstracts, 74 articles were excluded as duplicates. There were 95 articles unrelated to HCC or DKK-1 or diagnosis, 28 reviews, 19 abstracts, meeting reports or comments and replies and 9 papers on nonhumans; which were all excluded based on the inclusion and exclusion criteria. Subsequently, we read the full-texts of 16 articles, of which 8 articles^[22,23,26–31]^ exactly met the criteria for the meta-analysis, with adequate data for calculating SN and SP. These 8 studies included 3256 participants (1399 HCC and 1857 controls). The search process is shown in Figure 1. The included articles were published between 2011 and 2017. Among these articles, 10 studies of DKK-1,^[22,23,26–31]^ 9 studies of AFP^[22,23,26–29,31]^ and 7 studies of DKK-1 + AFP^[22,23,28,29,31]^ in HCC diagnosis were included. Elevated serum DKK-1 was found in HCC in all the studies. Among the 8 articles, 5 were conducted in China,^[22,23,26,29,30]^ while 3 in Korea, Turkey, and Egypt.^[27,28,31]^ All these diagnostic studies were retrospective, and the composition of the control group was different. Seven studies in 6 articles included cirrhosis, chronic hepatitis, or non-HCC liver disease alone as high-risk population into the control group in the HCC diagnosis by DKK-1,^[23,27–31]^ whereas 5 studies in 4 articles included high-risk population into the control group in the HCC diagnosis by DKK-1 + AFP.^[23,28,29,31]^ Six studies in 4 articles provided data on diagnosis of early HCC with DKK-1 and DKK-1 + AFP.^[22,23,28,29]^ Four studies in 3 articles provided data on distinguishing early HCC from high-risk control.^[23,28,29]^ The clinical features of the eligible articles are shown in Tables 1 and 2.

**Table 1:**
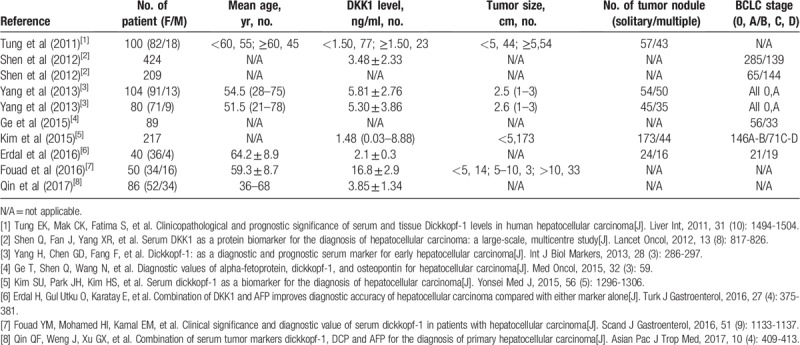
Characteristics of the studies included in the meta-analysis.

**Table 2:**
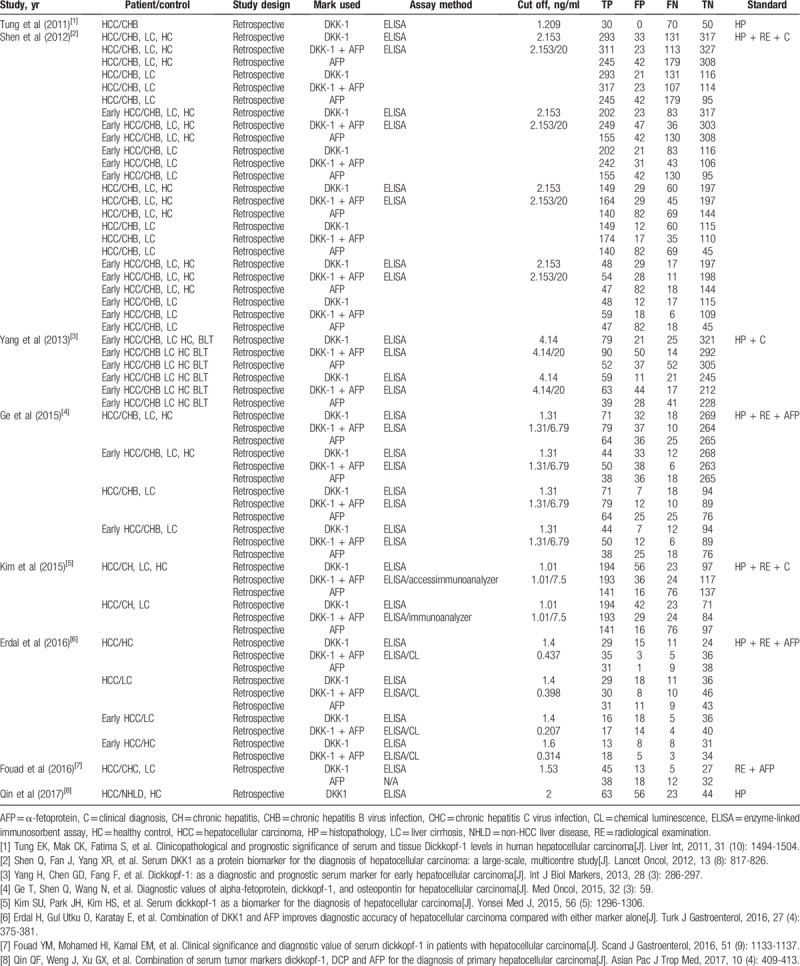
The diagnostic characteristics of DKK-1 in the included studies.

### 3.2 Quality assessment

**Figure 2:**
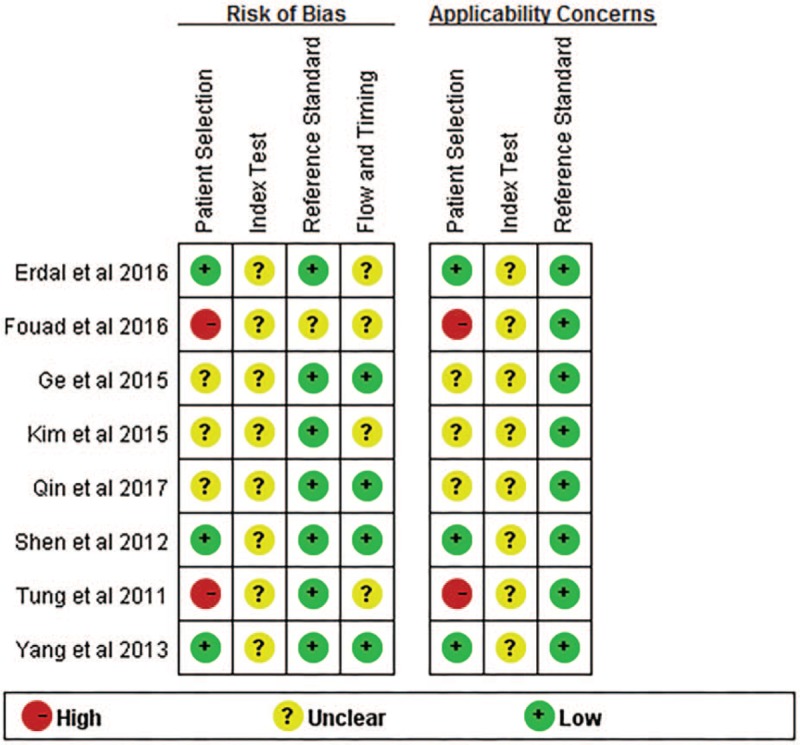
The quality of articles included.

The quality of 8 articles is demonstrated in Figure 2. In the patient selection domain, the risk of bias was noted as “unclear” for almost all included articles that did not illustrate whether consecutive or random patients were enrolled, except 3.^[22,23,28]^ The risk of bias was noted as “high risk” in patient selection domain for 3 studies as they only included high-risk population in the control.^[27,30]^ In the index test domain, the risk of bias was noted as “unclear” for the included articles without prespecified diagnostic thresholds, except 1.^[26]^ In the flow and timing domains, the risk of bias was noted as “low” for 4 articles as they used histopathology as reference standard for all included HCC,^[22,23,26,29]^ whereas it was noted as “unclear” for the remaining articles because they did not use the same reference standard for all included HCC.

### 3.3 Diagnostic accuracy

#### 3.3.1 Pooled diagnostic accuracy of DKK-1 in HCC diagnosis

**Figure 3:**
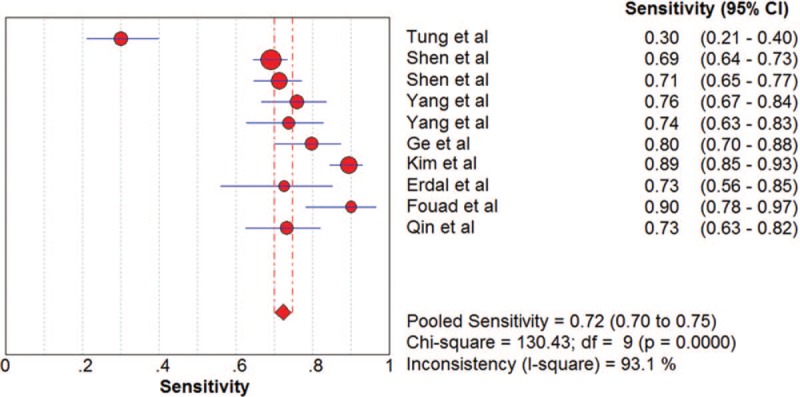
The pooled diagnostic accuracy of DKK-1 in HCC diagnosis. DKK-1 = dickkopf-1 protein, HCC = hepatocellular carcinoma.

**Figure 4:**
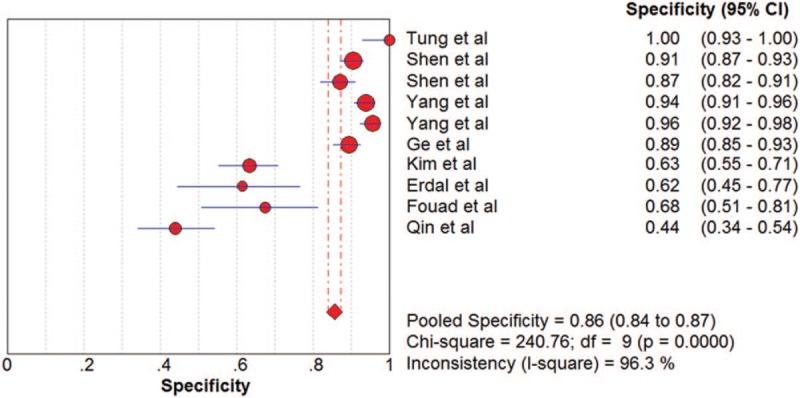
The pooled diagnostic accuracy of DKK-1 in HCC diagnosis. DKK-1 = dickkopf-1 protein, HCC = hepatocellular carcinoma.

**Figure 5:**
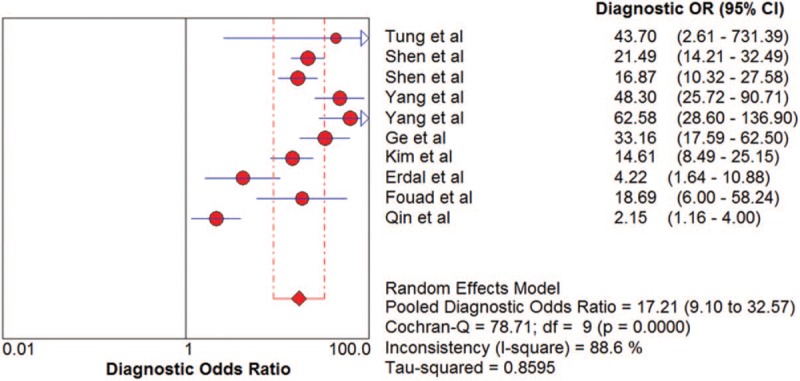
The pooled diagnostic accuracy of DKK-1 in HCC diagnosis. DKK-1 = dickkopf-1 protein, HCC = hepatocellular carcinoma.

**Figure 6:**
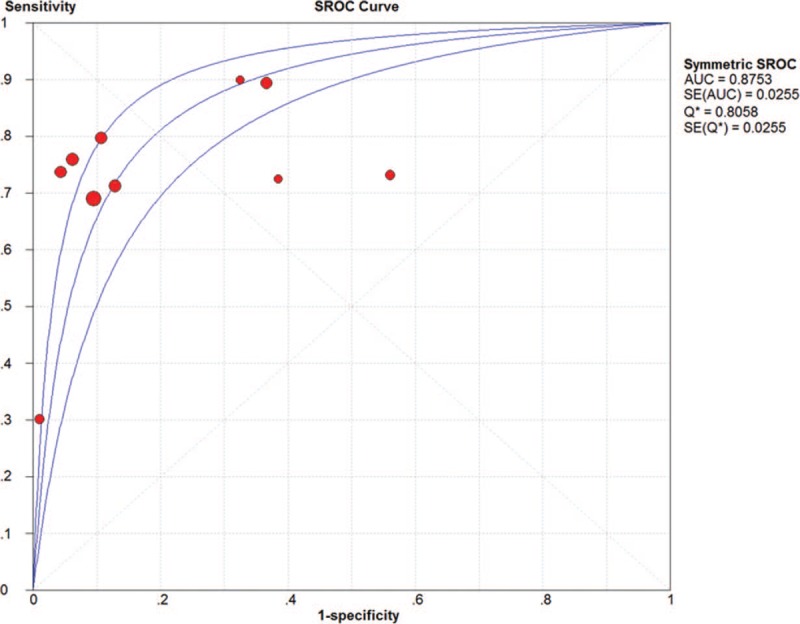
The pooled diagnostic accuracy of DKK-1 in HCC diagnosis. DKK-1 = dickkopf-1 protein, HCC = hepatocellular carcinoma.

The pooled SN and SP of DKK-1 in HCC diagnosis was 0.72 (95% CI: 0.70–0.75) and 0.86 (95% CI: 0.84–0.87), with *I*^2^ values of 93.1% and 96.3%. A bivariate random-effect model was executed due to the existence of substantial heterogeneity. The pooled PLR, NLR, and DOR were 4.91 (95% CI: 2.73–8.83), 0.32 (95% CI: 0.22–0.47), and 17.21 (95% CI: 9.10–32.57) with *I*^2^ values of 96.1%, 94.6%, and 88.6%, respectively. The sROC curve was plotted, and the area under curve (AUC) was 0.88 (SE = 0.0255) (Figs. 3–6). To analyze the source of heterogeneity, we first calculated threshold effects. The Spearman correlation coefficient between the logit of SN and the logit of 1-SP was 0.309 (*P* = .385), which indicated that the threshold effect did not result in heterogeneity among included studies. Consequently, subgroup analyses were performed to identify the potential sources of heterogeneity.

### 3.4 Subgroup analyses

Three subgroup analyses were conducted according to the stage of HCC and high-risk participants (patients with risk factors for HCC). The first analysis based on the composition of control group included those studies with high-risk population as control. Another analysis calculated diagnostic performance of DKK-1 for early HCC. The last subgroup analysis calculated diagnostic performance of DKK-1 for distinguishing early HCC from high-risk control (Table 3).

**Table 3:**
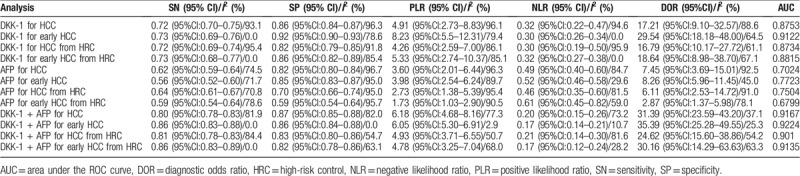
Summary of diagnostic accuracy of DKK-1 and DKK-1 + AFP.

A total of 1751 patients comprising of 1129 HCC patients and 622 patients with high-risk factors in 7 studies in 6 articles were identified,^[23,27–31]^ and the results were pooled as follows: SN was 0.72 (95% CI: 0.69–0.74), SP was 0.82 (95% CI: 0.79–0.85), DOR was 16.79 (95% CI: 10.17–27.72), and AUC was 0.87. The *I*^2^ values of SN, SP, and DOR were 95.4%, 91.8%, and 61.1%, respectively (Table 3).

A total of 2115 patients comprising of 611 early HCC patients and 1504 controls in 6 studies of 4 articles were identified,^[22,23,28–30]^ and the results were pooled as follows: SN was 0.73 (95% CI: 0.69–0.76), SP was 0.92 (95% CI: 0.90–0.93), DOR was 29.54 (95% CI: 18.18–48.00), and AUC was 0.91. The *I*^2^ values of SN, SP, and DOR were 0.0%, 78.6%, and 64.5%, respectively.

A total of 846 patients comprising of 427 early HCC patients and 419 high-risk participants in 4 studies of 3 articles were identified,^[23,28,29]^ and the results were pooled as follows: SN was 0.73 (95% CI: 0.68–0.77), SP was 0.86 (95% CI: 0.82–0.89), DOR was 18.64 (95% CI: 8.98–38.70), and AUC was 0.88. The *I*^2^ values of SN, SP, and DOR were 0.0%, 85.4%, and 67.1%, respectively.

### 3.5 Pooled diagnostic accuracy of AFP in HCC diagnosis

The pooled SN and SP of AFP in HCC diagnosis was 0.62 (95% CI: 0.59–0.64) and 0.82 (95% CI: 0.80–0.84), with *I*^2^ values of 74.5% and 96.7%. A bivariate random-effect model was executed due to the existence of substantial heterogeneity. The pooled PLR, NLR, and DOR were 3.60 (95% CI: 2.01–6.44), 0.49 (95% CI: 0.40–0.60), and 7.45 (95% CI: 3.69–15.01) with *I*^2^ values of 96.3%, 84.7%, and 92.5%, respectively. The sROC curve was plotted, and the AUC was 0.70 (SE = 0.0484) (Table 3). To analyze the source of heterogeneity, we calculated threshold effects. The Spearman correlation coefficient between the logit of SN and the logit of 1-SP was −0.050 (*P* = .898), which indicated that the threshold effect did not result in heterogeneity among included studies. Consequently, subgroup analyses were performed to identify the potential sources of heterogeneity.

### 3.6 Subgroup analyses

Three subgroup analyses were conducted as previously described (Table 3).

A total of 1585 patients comprising of 1029 HCC patients and 556 high-risk patients in 6 studies of 5 articles were identified,^[23,27–29,31]^ and the results were pooled as follows: SN was 0.64 (95% CI: 0.61–0.67), SP was 0.70 (95% CI: 0.66–0.74), DOR was 6.11 (95% CI:2.53–14.72), and AUC was 0.75. The *I*^2^ values of SN, SP, and DOR were 70.8%, 95.0%, and 91.0%, which meant that the type of control group was not the source of heterogeneity.

A total of 2065 patients comprising of 590 early HCC patients and 1475 controls in 5 studies of 3 articles were identified,^[22,23,29]^ and the results were pooled as follows: SN was 0.56 (95% CI: 0.52–0.60), SP was 0.85 (95% CI: 0.83–0.87), DOR was 8.26 (95% CI: 5.96–11.45), and AUC was 0.77. The *I*^*2*^ values of SN, SP, and DOR were 71.7%, 94.7%, and 45.0%.

A total of 771 patients comprising of 406 early HCC patients and 365 high-risk participants in 3 studies of 2 articles were identified,^[23,28,29]^ and the results were pooled as follows: SN was 0.59 (95% CI: 0.54–0.64), SP was 0.59 (95% CI: 0.54–0.64), DOR was 2.87 (95% CI: 1.37–5.98), and AUC was 0.68. The *I*^*2*^ values of SN, SP, and DOR were 78.6%, 95.7%, and 78.1%.

### 3.7 Pooled diagnostic accuracy of DKK-1 + AFP in HCC diagnosis

**Figure 7:**
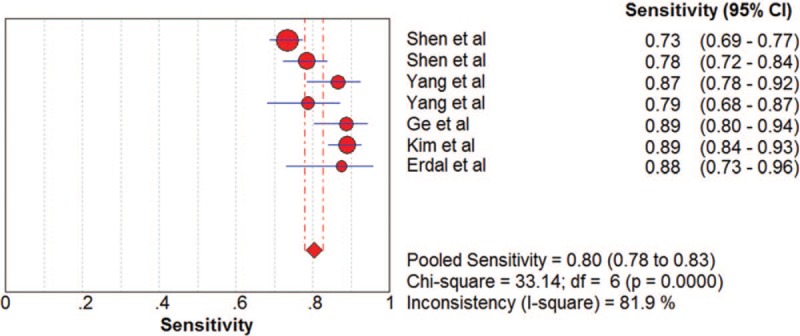
The pooled diagnostic accuracy of DKK-1 + AFP in HCC diagnosis. AFP = a-fetoprotein, DKK-1 = dickkopf-1 protein, HCC = hepatocellular carcinoma.

**Figure 8:**
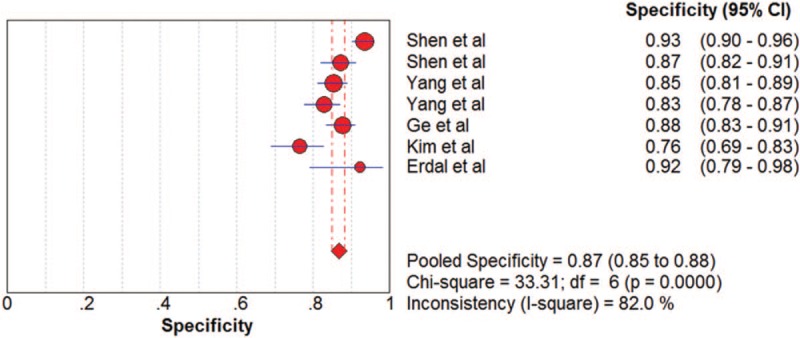
The pooled diagnostic accuracy of DKK-1 + AFP in HCC diagnosis. AFP = a-fetoprotein, DKK-1 = dickkopf-1 protein, HCC = hepatocellular carcinoma.

**Figure 9:**
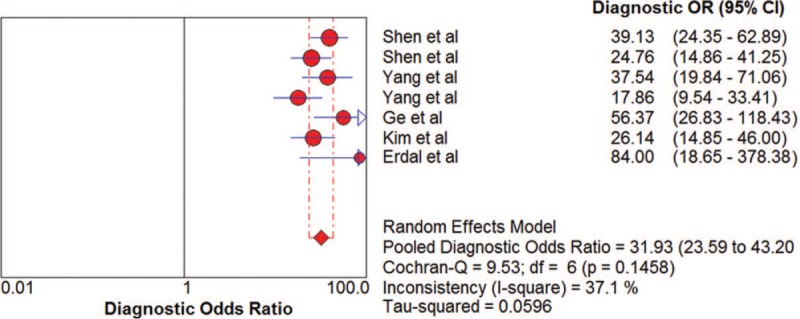
The pooled diagnostic accuracy of DKK-1 + AFP in HCC diagnosis. AFP = a-fetoprotein, DKK-1 = dickkopf-1 protein, HCC = hepatocellular carcinoma.

**Figure 10:**
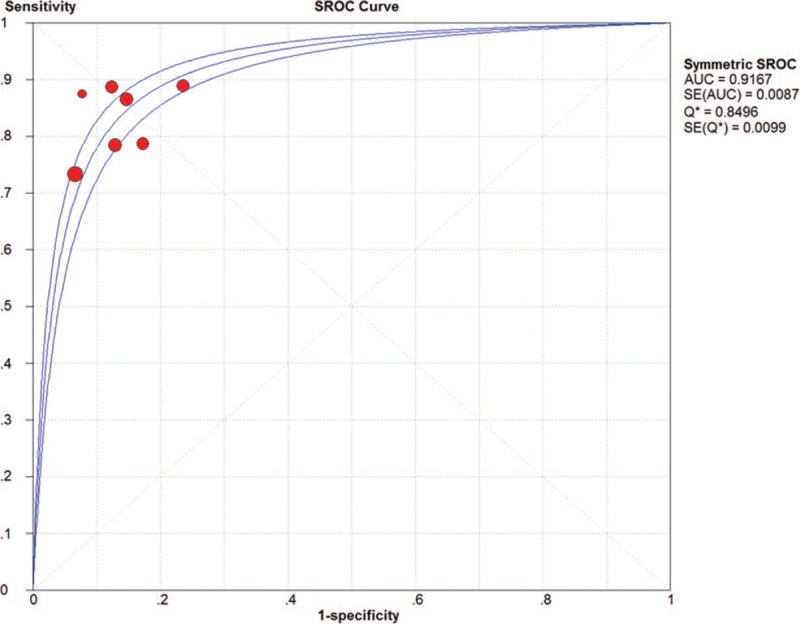
The pooled diagnostic accuracy of DKK-1 + AFP in HCC diagnosis. AFP = a-fetoprotein, DKK-1 = dickkopf-1 protein, HCC = hepatocellular carcinoma.

The pooled SN and SP of DKK-1 + AFP in HCC diagnosis was 0.80 (95% CI: 0.78–0.83) and 0.87 (95% CI: 0.85–0.88) with *I*^2^ values of 81.9% and 82.0%. A bivariate random-effect model was used due to the presence of substantial heterogeneity. The pooled PLR, NLR, and DOR were 6.18 (95% CI: 4.68–8.16), 0.20 (95% CI: 0.15–0.26), and 31.39 (95% CI: 23.59–43.20) with *I*^2^ values of 77.3%, 73.2%, and 37.1%, respectively (Figs. 7–10). The sROC curve was plotted, and AUC was 0.92 (SE = 0.0099). To analyze the source of heterogeneity, we first calculated threshold effects. The Spearman correlation coefficient between the logit of SN and the logit of 1-SP was 0.429 (*P* = .337), which meant that the threshold effect did not cause heterogeneity among the included studies. Consequently, subgroup analyses were performed to identify the potential sources of heterogeneity.

### 3.8 Subgroup analyses

Three subgroup analyses were conducted as previously described (Table 3).

A total of 1511 patients comprising of 979 HCC patients and 532 high-risk patients in 5 studies of 4 articles were identified,^[23,28,29,31]^ and the results were pooled as follows: SN was 0.81 (95% CI: 0.78–0.83), SP was 0.83 (95% CI: 0.80–0.86), DOR was 24.62 (95% CI:15.60–38.86), and AUC was 0.90. The *I*^*2*^ values of SN, SP, and DOR were 84.4%, 54.7%, and 54.2%, which meant that the type of control group was not the source of heterogeneity.

A total of 2125 patients comprising of 611 early HCC patients and 1504 controls in 6 studies of 4 articles were identified,^[22,23,28,29]^ and the results were pooled as follows: SN was 0.86 (95% CI: 0.83–0.88), SP was 0.86 (95% CI: 0.84–0.88), DOR was 35.39 (95% CI: 25.28–49.55), and AUC was 0.92. The *I*^*2*^ values of SN, SP, and DOR were 0.0%, 0.0%, and 25.3%.

A total of 846 patients comprising of 427 early HCC patients and 419 high-risk participants in 4 studies of 3 articles were identified,^[23,29]^ and the results were pooled as follows: SN was 0.86 (95% CI: 0.83–0.89), SP was 0.82 (95% CI: 0.78–0.86), DOR was 30.16 (95% CI: 14.29–63.63), and AUC was 0.91. The *I*^*2*^ values of SN, SP, and DOR were 0.0%, 63.1%, and 63.3%.

### 3.9 SN analysis and publication bias

**Figure 11:**
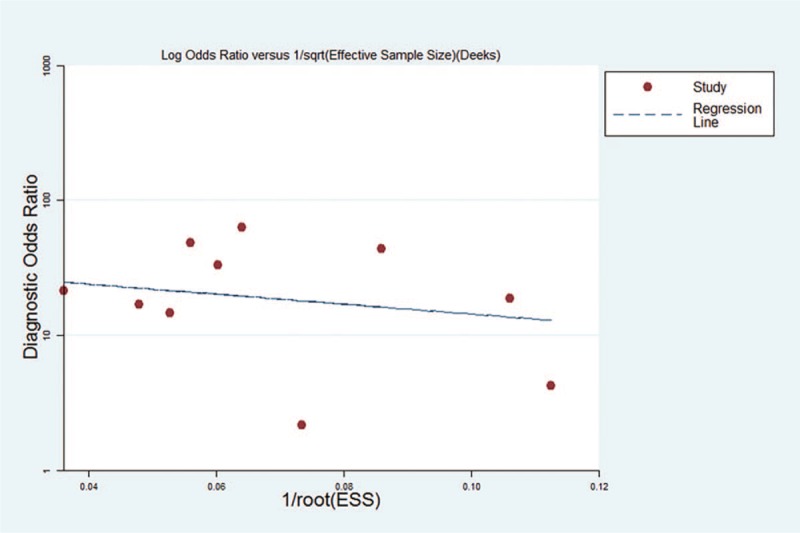
Deek's plots of the publication bias among all the included studies.

The SN analysis was performed to estimate the impact of each study in diagnosing HCC with DKK-1 alone and DKK-1 + AFP, and the result revealed that the data were stable. We used Deeks’ funnel plot asymmetry test to evaluate the publication bias, and the *P*-value was .585 (DKK-1 alone) and .693 (DKK-1 + AFP), which indicated no potential publication bias among all the included studies (Fig. 11).

## 4 Discussion

Given that cirrhotic hepatitis patients and chronic HBV carriers are recommended for regular surveillance to avoid the tumorigenesis of HCC, timely diagnosis of HCC provides more therapeutic options, and better prognosis for patients.^[2]^ In case histopathology data is unavailable, serum AFP level combined with medical imaging could be used to detect HCC.^[5]^ However, low SN of AFP makes it a sub-optimal marker for HCC screening and in 5% to 7% cases, imaging could not distinguish HCC from other non-HCC tumors.^[39,40]^ Hence, it is critical to search for a biomarker that can complement AFP or even replace it, and act as a reliable index.

In this meta-analysis, we extracted adequate data for calculating SN, SP, PLR, NLR, DOR, and AUC of sROC from 8 articles that performed diagnostic tests for detecting HCC with serum DKK-1 alone, AFP alone, and DKK-1 + AFP. The pooled results were 0.72, 0.62, and 0.80; 0.86, 0.82, and 0.87; 4.91, 3.60, and 6.18; 0.32, 0.49, and 0.20; 17.21, 7.45, and 31.93; 0.88, 0.70, and 0.92, respectively. DKK-1 alone showed good accuracy in HCC diagnosis, while DKK-1 + AFP showed even better accuracy with DOR of 31.93, AUC of 0.92. However, AFP owned the worst diagnostic efficacy when compared to DKK-1 or the combination of DKK-1 and AFP, as revealed in Table 3. Although a previous meta-analysis has examined this issue,^[32]^ the present analysis deserves attention, because more studies were included and 2 different subgroup analyses were conducted. During the process of screening potential eligible studies, we set the inclusion and exclusion criteria similar to the previous meta-analysis and thus 2 studies that used plasma as the sample to explore the diagnostic value of DKK-1 in HCC diagnosis were excluded.^[24,25]^ It was also a consideration of homogeneity because biomarkers examined by different samples were in very dynamic concentrations.^[41]^ There was no limitation in language of included articles in the previous meta-analysis, but in the present meta-analysis, only articles published in English were included. As compared to the results of the previous meta-analysis, the AUC was 0.88 versus 0.84, while the DOR had decreased more than one-third (17.21 vs 26.90) in the present meta-analysis, which indicated that serum DKK-1 alone may not be optimal in diagnosing HCC. For the combination of DKK-1 and AFP, the AUC was 0.92 versus 0.88 and the DOR was 31.93 versus 24.60 in the current and previous meta-analysis, which indicated that DKK-1 + AFP was more suitable for HCC diagnosis than DKK-1 alone.

Serum DKK-1 had shown diagnostic value in diagnosing HCC in many studies,^[22–31]^ and majority of them concluded that DKK-1 could commendably detect HCC, except Mao et al to differentiate AFP (−) HCC from liver cirrhosis.^[24]^ In another study published in 2012, serum DKK-1 showed a moderate diagnostic value in distinguishing AFP (−) HCC from high-risk patients.^[23]^ However, it is difficult to predict whether DKK-1 could display a good diagnostic accuracy in AFP (−) HCC as there was insufficient data to analyze in the current meta-analysis. Thus, more studies on diagnosing AFP (−) HCC with serum DKK-1 are needed.

The previous meta-analysis conducted by Zhang et al indicated that both DKK-1 and DKK-1 + AFP had satisfactory accuracy for diagnosing HCC,^[32]^ with the pooled SN of 0.65 and 0.81, SP of 0.94 and 0.85, and AUC of 0.84 and 0.88. As compared to the above results by Zhang et al, others markers utilized for diagnosing HCC showed different diagnostic accuracy in the same year (2014). The meta-analysis of osteopontin (OPN), glypican-3 and des-γ-carboxy prothrombin (DCP) in 2014 demonstrated the following results^[42–44]^: SN were 0.88, 0.53, and 0.71, respectively. SP were 0.87, 0.77, and 0.84, respectively. AUC were 0.91, 0.82, 0.89, respectively. The corresponding updated meta-analysis showed 0.71, 0.80, and 0.8786 for OPN, 0.68, 0.92, 0.87 for GCP3, 0.69, 0.89, and 0.88 for DCP.^[45–47]^ As compared to the results pooled in meta-analysis of different markers, our results with SN of 0.72, SP of 0.86 and AUC of 0.8596 in HCC diagnosis with serum DKK-1 alone might seem moderate. However, with AUC of 0.92, the combination of DKK-1 and AFP showed an equivalent diagnostic performance as compared to OPN and DCP.^[46,47]^

Heterogeneity among the included studies was evaluated through different methods in this analysis since it is an indicator of the reliability of the results. Threshold effect was thought to be a primary cause for heterogeneity in diagnostic studies. In the current meta-analysis, the Spearman correlation coefficients of DKK-1 alone, AFP alone, and DKK-1 + AFP in diagnosing HCC were 0.378 (*P* = .226), −0.050 (*P* = .898), and 0.119 (*P* = .779), which indicated that threshold effect did not exist as all *P*-values were >.05. Then, we performed subgroup analyses according to the stage of HCC and high-risk control in DKK-1 alone, AFP alone, and DKK-1 + AFP, respectively. The *I*^*2*^ values of DOR in 3 subgroups of DKK-1 were 61.1%, 64.5%, and 67.1%, respectively. The *I*^*2*^ values of DOR in 3 subgroups of AFP were 91.0%, 45.0%, and 78.1%, respectively. The *I*^2^ values of DOR of DKK-1 + AFP were 54.2%, 25.3%, and 63.3% (Table 3). As compared to the *I*^2^ value (37.1%) of DOR of DKK-1 + AFP in diagnosing all HCC patients, we found that the stage of HCC was the source of heterogeneity, as the *I*^*2*^ value of DOR decreased >10% and the *I*^2^ values of both pooled SN and SP were 0.0%. Similar to the previous meta-analysis, *I*^2^ value of pooled SN in DKK-1 alone of early HCC subgroup was 0.0%, which indicated the stage of HCC was the source of heterogeneity in the current meta-analysis. However, the stage of HCC failed to appropriately explain the potential source of heterogeneity of SP in DKK-1 alone, even though the *I*^*2*^ values of DOR decreased >10%. Likewise, all of them were still >50%.

The limitations in the included studies and this meta-analysis were as follows:

(1) As compared to the previous meta-analysis, the overall participants in the diagnosis test did not significantly increase, although more studies were included in the current meta-analysis (2678 vs 1115). However, 2 large samples were predominantly included in the previous meta-analysis,^[22,23]^ which might lead to bias of the result. Hence, it is reasonable and necessary to further confirm the diagnostic performance of DKK-1 and DKK-1 + AFP.
(2) The study design of all included studies was retrospective, and poor results might be removed from raw data. Besides, the purpose of included studies was incongruous.
(3) There were only 3 studies with non-Chinese blood samples,^[27,28,31]^ and only articles published in English were screened, which may have led to the limitations of geographical regions and languages.
(4) Different cut-off values of serum DKK-1 were used among the included studies, which made it difficult to estimate the real diagnostic value. However, as a novel marker, DKK-1 should be tested for detecting HCC in future studies to explore the optimum cut-off value.
(5) The standard references of HCC diagnosis differed among the included studies, including biochemistry, imaging characteristics, and pathology. However, it is difficult to have uniform methods for diagnosis of diseases in clinical practice.
(6) Due to constraints of the small number of included studies, we did not perform meta-regression in the current meta-analysis to further search for the source of heterogeneity. Although subgroup analyses identified that the stage of HCC was the source of heterogeneity of DKK-1 + AFP in HCC diagnosis, it could not confirm whether the stage of HCC was the source of heterogeneity in DKK-1.

## 5 Conclusion

Serum DKK-1 + AFP showed high accuracy for diagnosing HCC, while serum DKK-1 alone, with a lower DOR, showed moderate accuracy as compared to the previous meta-analysis. However, more studies are needed to ascertain the diagnostic value of serum DKK-1 in AFP (−) HCC. Due to the limitations of the current meta-analysis, further well-designed studies are needed to confirm the diagnostic value of DKK-1 and DKK-1 + AFP in HCC diagnosis.

## Author contributions

**Data curation:** Yi Zeng, Xueyi Tang.

**Formal analysis:** Zhenjie Li, Zuhui Pu.

**Investigation:** Lisha Mou, Yi Zeng, Xueyi Tang.

**Methodology:** Zhenjie Li, Yongqiang Zhan.

**Resources:** Xueyi Tang.

**Software:** Zhenjie Li, Lisha Mou, Haibin Gao, Yi Zeng.

**Supervision:** Xuesong Deng, Yongqiang Zhan.

**Validation:** Zhenjie Li, Haibin Gao, Xueyi Tang, Zuhui Pu, Yong Ni.

**Writing – original draft:** Zhenjie Li.

**Writing – review and editing:** Xuesong Deng.
