# Diagnostic accuracy of serum dickkopf-1 protein in diagnosis hepatocellular carcinoma: an updated meta-analysis: Erratum

**DOI:** 10.1097/MD.0000000000017062

**Published:** 2020-02-07

In the article, “Diagnostic accuracy of serum dickkopf-1 protein in diagnosis hepatocellular carcinoma: An updated meta-analysis”,^[1]^ which appears in Volume 98, Issue 32 of *Medicine*, the authors would like to note that the project was supported by the Shenzhen Committee of Science and Technology, China (Grant No. JCYJ20160425103911638).
